# Examining E-Loyalty in a Sexual Health Website: Cross-Sectional Study

**DOI:** 10.2196/publichealth.5393

**Published:** 2017-11-02

**Authors:** Alexandra Nunn, Rik Crutzen, Devon Haag, Cathy Chabot, Anna Carson, Gina Ogilvie, Jean Shoveller, Mark Gilbert

**Affiliations:** ^1^ Clinical Prevention Services British Columbia Centre for Disease Control Vancouver, BC Canada; ^2^ Department of Health Promotion CAPHRI School for Public Health and Primary Care Maastricht University Maastricht Netherlands; ^3^ School of Population and Public Health University of British Columbia Vancouver, BC Canada; ^4^ Women's Health Research Institute BC Women's Hospital and Health Centre Vancouver, BC Canada

**Keywords:** e-loyalty, sexual health, Internet, patient satisfaction, cross-sectional studies

## Abstract

**Background:**

Web-based sexual health resources are typically evaluated in terms of their efficacy. Information is lacking about how sexual health promotion websites are perceived and used. It is essential to understand website use to address challenges with adherence and attrition to Web-based health interventions. An existing theoretical framework for examining loyalty to electronic health (eHealth) interventions has been not yet been applied in the context of sexual health promotion nor has the association between e-loyalty and intended intervention efficacy outcomes been investigated.

**Objective:**

The objectives of this study were to investigate users’ loyalty toward a sexual health website (ie, e-loyalty), measure user perceptions of the website, and measure the association between e-loyalty and perceived knowledge increase and intent to change behavior.

**Methods:**

Over 4 months, website users (clients and health care providers) participated in an open, online, cross-sectional survey about their user experiences that measured e-loyalty, user perceptions, and intended website efficacy outcomes. Relationships between user perceptions and e-loyalty were investigated using structural equation modeling (SEM). Associations between e-loyalty and website efficacy outcomes were tested using Spearman rank correlation.

**Results:**

A total of 173 participants completed user perception questions and were included in the analysis. E-loyalty was high for both clients and providers and was significantly correlated with clients’ perceived knowledge increase (ρ(171)=.30, *P*<.001), their intent to have safer sex (ρ(171)=.24, *P*=.01), and their intent to get tested for sexually transmitted infections (ρ(171)=.37, *P*<.001). The SEM showed that trustworthiness, overall experience, active trust, and effectiveness were directly related to e-loyalty. Finding the website “easy to understand” was significantly related to active trust (ie, participants’ willingness to act upon information presented on the website).

**Conclusions:**

E-loyalty may be related to the efficacy of the selected website in improving one’s sexual health and was significantly associated with all three intended knowledge and behavioral outcomes. To increase e-loyalty, trustworthiness and active trust are important user perceptions to deliberately engender. Our findings indicate that understanding a website contributes to active trust, thereby highlighting the importance of considering eHealth literacy in designing health promotion websites. Our study confirms the relevance of e-loyalty as an outcome for evaluating the antecedents of the use and efficacy of online public health interventions across disciplines by adapting and validating an existing e-loyalty framework to the field of sexual health promotion. Our findings suggest that e-loyalty is positively associated with measures of website efficacy, including increased knowledge and intent to change behavior. Longitudinal research with larger samples could further investigate the relationships between e-loyalty, website understandability, and outcomes of online health interventions to determine how the manipulation of website characteristics may impact user perceptions and e-loyalty.

## Introduction

### Background

Web-based resources for sexual health promotion, including the resources on prevention of sexually transmitted infections (STIs) and human immunodeficiency virus (HIV), have been accessible for over 15 years, by virtue of the advantage of the Internet to deliver personal or tailored sexual health information to large audiences [1]. Web-based sexual health resources are easily accessible, can be accessed anonymously, and can be visited repeatedly and at convenient times [2,3]. Although barriers to accessing Web-based sexual health interventions (eg, low income, low education or geographic remoteness) persist in Canada, the digital divide is continuously closing [4]. Increasingly, focus is turning to Web-based sexual resources to reach groups at high risk for STIs and HIV, such as youth and men who have sex with men (MSM) [5] as Web-based resources can uniquely provide sexual health information that is relevant and free of prejudice to users regardless of their gender, age, sexual orientation, and location [6,7].

Web-based sexual health resources are typically evaluated in terms of their efficacy, for example, using randomized controlled trials to assess self-reported outcomes before and after exposure to the intervention [8,9]. However, a new pragmatic field of research has been emerging to focus on the *use* of health interventions, which seeks to understand why individuals in the general public choose to engage with and remain loyal to a particular website [10]. Studying website use is particularly important because attrition and low usage are fundamental challenges to Web-based interventions and the low threshold to participate makes it easy for users to leave [11]. E-loyalty, or loyalty to a website, is a well-described concept in e-commerce that pertains to users’ behavioral intent, such as the intention to buy a product online from one website rather than another or the intention to return to a website in the future [12]. Models of the cognitive elements related to e-loyalty from the e-commerce field have recently been applied and validated in the field of eHealth [13,14]. Understanding how users’ perceptions of a website contribute to e-loyalty is important for the design of websites and for establishing trust and eliciting repeat visits with the objective of delivering impactful public health interventions that ultimately will lead to better health outcomes for users [13,14].

A theoretical framework to conceptually define e-loyalty and its antecedents for public health interventions was developed by Crutzen et al (2011) and has been applied to websites in health domains, including cancer patient education, mental health and addictions, and injury prevention [13]. This study applies the e-loyalty framework to sexual health, based on a Canadian provincial sexual health website that is developed and managed by the British Columbia Centre for Disease Control (BCCDC). SmartSexResource (SSR) is an open website (ie, no username or password required) with both interactive and static features, comprising content for both individuals seeking sexual health information (clients) and health care professionals (providers) [15]. Users can access pages for specific topics, input their postal code or city and search for clinics based on their services and opening hours, and interact with a health care provider by chatting with a nurse during specified hours or by submitting an anonymous question to which a nurse responds in a private email or via a public posting. One of the main objectives of SSR is to increase visitors’ satisfaction with sexual health services in British Columbia and improve the visitor experience by engendering perceptions of confidentiality and trustworthiness—concepts well aligned with the e-loyalty framework.

### Theoretical Framework

The study employed an e-loyalty framework for health interventions, put forth by Crutzen et al, which involves measuring user perceptions of a website that contribute to the outcome measures of e-loyalty (ie, intention to visit the site again and recommending a website to others). The e-loyalty concept maintains that a positive user experience leads to increased website use [13]. See Table 1 for the constructs that comprise the e-loyalty framework.

Given the sensitive and personal nature of sexual health topics, and the fact that visitors to SSR often submit questions that are motivated by uncertainty or fear and related to personal sexual experiences, we modified the e-loyalty framework by removing a previously validated component called “enjoyment” and substituting a question to reflect the overall experience of the website.

Furthermore, our study included a measure of visitors’ understanding of the website, acknowledging the importance of considering eHealth literacy in the online presentation of health information. The term “eHealth literacy” is defined as “the ability to seek, find, understand, and appraise health information from electronic sources and apply the knowledge gained to addressing or solving a health problem” [16]. Existing components of the e-loyalty framework can be considered to reflect eHealth literacy (eg, the questions about efficiency and effectiveness measure “information literacy,” one of the six components of eHealth literacy, which involves the ability to navigate resources, search strategically, and filter results to find relevant information). Therefore, we decided to include “understanding” as an additional user perception in our analysis of e-loyalty. Understanding was measured on a 7-point Likert scale, in which respondents rated their agreement with the statement, “I found the information easy to understand.”

In summary, we sought to measure the e-loyalty of visitors to SSR and determine how the e-loyalty framework applies to sexual health promotion. We also explored how understanding fits within the e-loyalty framework and whether e-loyalty was associated with self-reported knowledge and behavior changes following the use of SSR.

## Methods

### Study Design and Recruitment

We administered a cross-sectional online survey to SSR visitors between April and August 2014. The aim of the survey was to learn about the experiences and perceptions of visitors who use SSR as a resource for Web-based sexual health information. Participants were recruited either from a banner ad on the SSR homepage or through an email invitation to health care providers who subscribed to an SSR email distribution list. The survey was an open survey, available to all visitors of the SSR website regardless of their location [17]; however, given the focus of SSR on providing information relevant to Canadians and the residents of British Columbia in particular, we provided an incentive to oversample Canadian participants (entered into a draw for one of two mini tablet computers if they had a Canadian postal code).

### Survey Description

The user perception questions were located midway through the 31-item survey, with demographic questions at the end. At the start, participants were classified by visitor type (either “client” or “provider”) based on their response to initial questions assessing whether they were visiting SSR for work or for personal reasons. Adaptive questioning was used to tailor questions to each group. Although prior e-loyalty research has employed 2 to 3 items per user perception, we used a single item per user perception, given the internal structure in prior research and the conceptual robustness of the user perceptions that have now been validated in multiple eHealth fields, as well as a desire to minimize survey length [13,14].

The user perception questions were followed by 3 questions pertaining to behavioral and knowledge outcomes of visiting the website. Clients and providers both reported whether their knowledge about STIs or sexual health had increased as a result of visiting SSR, and only clients reported whether they were more likely to have safer sex and get tested for HIV and STIs. All of the aforementioned outcomes were measured by presenting participants with a statement to which they reported their level of agreement on a 7-point Likert scale (strongly disagree to strongly agree). The survey questions included in this study are described in Table 1.

**Table 1:** E-loyalty user perceptions.

Model		Explanation	Survey item^a^
**User perceptions derived from Crutzen et al [10]**		
	Intention to visit again (return)		I would use this website again.
	Recommend to others		I would recommend this website to others.
	Effectiveness	Quality and relevance of the information	The website provided me with relevant information about sexual health.
	Efficiency	Easy search of and access to information	I was able to access the information quickly on this website.
	Trustworthiness	Believability of the provided information	I trusted the information presented on this website.
	Active trust	Confidence in acting on the provided information	I would act upon the information presented on this website.
**User perceptions added in this study**		
	Overall experience	Positive perception of the website	Based on today’s visit, how would you rate your SSR experience overall? (7-point Likert scale from very poor to very good)
	Understanding		I found the information easy to understand.
**Behavioral and knowledge outcomes**		
	Knowledge increase		As a result of visiting SmartSexResource, my knowledge about STIs or sexual health has increased.
	Behavioral intent #1		As a result of visiting SmartSexResource, I am more likely to have safer sex.
	Behavioral intent #2		As a result of visiting SmartSexResource, I am more likely to get tested for HIV^b^ or STIs^c^.

^a^Unless otherwise specified, the question was answered on a 7-point Likert scale from strongly disagree (1) to strongly agree (7).

^b^HIV: human immunodeficiency virus.

^c^STIs: sexually transmitted infections.

**Table 2:** Sample characteristics for SmartSexResource visitor survey 2014, n=173.

Characteristic		Clients (N=131)	Providers (N=42)	Total
		n (%)^b^	n (%)^b^	n (%)^b^
**Age, in years**				
	<20	16 (15)	1 (3)	17 (12)
	20-29	41 (38)	8 (21)	49 (34)
	30-39	22 (21)	10 (26)	32 (22)
	40-49	11(10)	10 (26)	21 (14)
	>50	17 (16)	9 (24)	26 (18)
	Total^a^	107	38	145
**Gender identity**				
	Female (woman)	55 (58)	31 (82)	86 (65)
	Male (man)	38 (40)	5 (13)	43 (32)
	Transgender	2 (2)	0 (0)	2 (2)
	Genderqueer	0 (0)	2 (5)	2 (2)
	Total^a^	95	38	133
**Education**				
	Primary	5 (5)	0 (0)	5 (3)
	Secondary	28 (26)	1 (3)	29 (20)
	College or university	59 (55)	21 (54)	80 (54)
	Graduate level	16 (15)	17 (43)	33 (22)
	Total^a^	108	39	147
**Location**				
	Canada	97 (74)	39 (95)	136 (79)
	International	34 (26)	2 (5)	36 (21)
	Total^a^	131	41	172

^a^Total represents the data available (ie, excluding missing values) or number of respondents to the survey question.

^b^Percentage is the proportion of respondents who answered the survey question (ie, excluding missing values).

The survey was pilot-tested and revised to make the majority of questions optional. Mandatory questions were limited to 2 questions that oriented clients and health care providers to slightly different survey streams (eg, providers were not asked about their intentions to change sexual health behaviors) [17].

Additional survey domains included frequency and purpose of visiting SSR, use of other sexual health information sources, use of or preference for particular features of SSR, and sexual identity.

### Analysis

We used chi-squared tests (Fisher exact tests where cell counts were less than 5) to determine significant differences between respondents who completed the e-loyalty section and those who did not (and thus were excluded from further analysis). Respondents’ location was determined by triangulating data from the Fluidsurveys software, Piwik Open Analytics Platform website metrics software, and respondents’ self-reported location.

Summary measures of user perceptions were reported using the mean to describe the average rating on a 7-point Likert scale. Differences in e-loyalty and user perceptions by respondent characteristics were determined by the Wilcoxon rank-sum test as the data were non-normal. Accordingly, associations between e-loyalty and knowledge and behavioral intent outcomes were measured using a nonparametric measure of association, Spearman ρ (rho; two-tailed).

The relationships between user perceptions and e-loyalty were investigated through structural equation modeling (SEM), using MPlus V7.2 software (Muthén & Muthén) . Covariates in model building included education, gender, and visitor type (ie, client or provider). The model was built to optimize fit indices, including the comparative fit index (CFI), Tucker-Lewis Index (TLI), and root mean squared error of approximation (RMSEA). Because the two e-loyalty outcomes “recommend to others” and “intention to visit again” were highly correlated (ρ(171)=.77, *P*<.001), we used the mean of the two outcome measures to represent overall e-loyalty.

## Results

### Respondents

During the 4-month study period, the mean number of unique visitors per day was 879 (range: 571-1209), of which the mean number of returning visitors was 146 per day (range: 80-227). In total, 501 unique survey responses were received. Of those, 37 responses (7.4%; 37/501) were from Internet Protocol (IP) addresses associated with two or more survey responses. The majority of respondents (96.4%; 483/501) accessed the survey through a banner ad on SSR; the rest (3.6%; 18/501) followed an email link. In total, 173 respondents (34.5%; 173/501) completed all e-loyalty questions and were included in the analysis. Participants completing the e-loyalty questions were more likely to be health care providers (67% [42/63] vs 30.0% [131/438] of clients, n=501, χ^2^_1_=31.3, *P*<.001), self-reported returning visitors to the site (73% [52/71] vs 45.6% [121/265] of first-time visitors, n=336, χ^2^_1_=17.1, *P*<.001), female or woman (99% [86/87] vs 78% [47/60] of other gender identities, n=147, χ^2^_1_=17.4, *P*<.001), and Canadians (47.4% [136/287] vs 17.1% [36/210] of non-Canadians, n=497, χ^2^_1_=49.0, *P*<.001). Completion of the e-loyalty section was not significantly associated with age or level of education. The final sample for the analysis comprised 131 clients (75.7%; 131/173) and 42 health care providers (24.3%; 42/173), of which 30.1% (52/173) in total were returning visitors to the site. The sample of clients represented youth younger than 20 years (15.0%; 16/107) and MSM (10.8%; 12/111, data not shown)—high-risk groups that are key target audiences for the website. For a description of participants, see Table 2.

### E-Loyalty Outcome

The average e-loyalty score was high for both clients (mean 5.62) and providers (mean 6.52). Visitors returning to the site had higher mean e-loyalty scores than first-time visitors (6.48 vs 5.61, n=170, W=1390.5, *P*<.01). For clients and providers combined, the highest rated user perception was “understanding,” with a mean rating of 6.01, followed by “trustworthiness” (mean: 5.99) and “active trust” (mean: 5.97). Providers consistently gave higher ratings for all user perceptions and both e-loyalty measures (Table 3). Among clients and providers combined, e-loyalty was most highly correlated with active trust (ρ=.79) and trustworthiness (ρ=.79), followed by understanding (ρ=.69). See Table 4.

### Knowledge and Behavior Outcomes

After visiting the site, 71% of respondents reported that their knowledge increased. The majority of clients reported that after visiting SSR, they were more likely to have safer sex (58%) and get tested for HIV and STIs (61%); health care providers were not asked these 2 behavioral questions. All three outcome measures were significantly associated with higher e-loyalty scores. For all respondents, e-loyalty was positively correlated with perceived knowledge increase (ρ(171)=.30, *P*<.001). For clients, e-loyalty was positively correlated with both intent to have safer sex (ρ(171)=.24, *P*=.01) and intent to get tested for STIs (ρ(171)=.37, *P*<.001).

### User Perceptions and E-Loyalty

**Figure 1:**
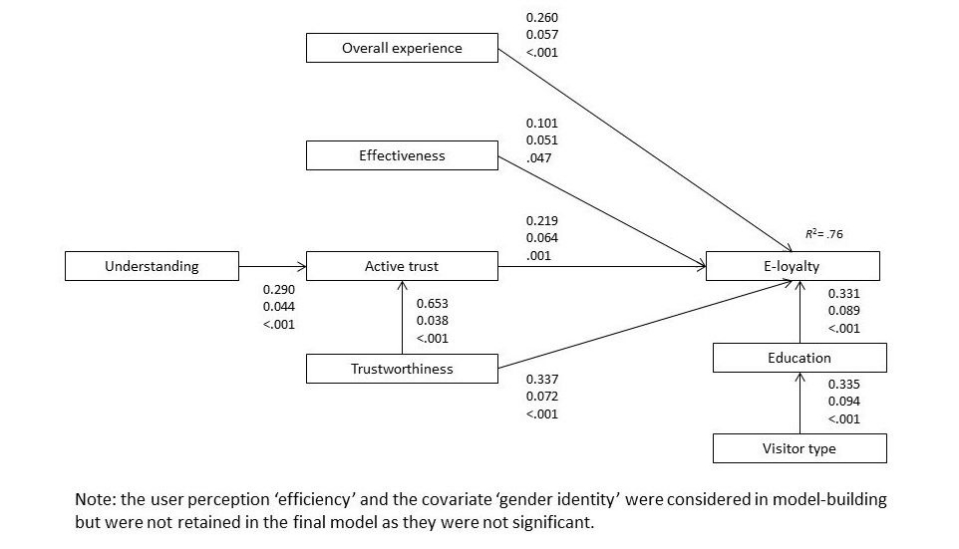
Structural equation model for the relationships between five user perceptions and e-loyalty. (Note: Numbers next to paths indicate standardized estimates, standard errors, and P values).

Our SEM tested the relationships between user perceptions and e-loyalty, the effect of understanding and trustworthiness on active trust, and the effect of gender, visitor type, and education on e-loyalty. The final model had adequate acceptable fit according to fit indices, with a CFI of 0.95, TLI of 0.92, and RMSEA of 0.06 (90% CI 0.03-0.09) [18]. The SEM developed to identify relationships between user perceptions and e-loyalty is shown in Figure 1 (output is shared in Multimedia Appendix 1). The variance in e-loyalty was well explained by the model (*R*^2^=.76).

**Table 3:** Summary of outcome and user perception ratings for SmartSexResource visitor survey 2014.

Measure		Mean rating by visitor type (scale: 1-7^a^)		
		Client (n=131)	Provider (n=42)	*P* value
**Outcomes**				
	E-loyalty	5.62	6.52	<.001
	Return	5.78	6.51	<.001
	Recommend	5.50	6.54	<.001
**User perceptions**				
	Overall	5.59	6.14	.004
	Effectiveness	5.78	6.17	.008
	Efficiency	5.73	5.98	.15
	Understanding	5.85	6.50	<.001
	Trustworthiness	5.78	6.64	<.001
	Active trust	5.79	6.52	<.001

^a^Scale: 1=strongly disagree, 2=disagree, 3=somewhat disagree, 4=neutral, 5=somewhat agree, 6=agree, 7=strongly agree.

**Table 4:** Correlation matrix and mean user perception ratings, clients and providers (n=173).

Measure		Mean (range: 1-7)	SD^a^	1	1a	1b	2	3	4	5	6	7
**1. E-loyalty**		5.84	1.28	-	.90	.96	.61	.56	.56	.72	.79	.79
	a. Return	5.96	1.25		-	.77	.63	.56	.57	.67	.71	.73
	b. Recommend	5.74	1.39			-	.56	.54	.52	.70	.81	.77
2. Overall experience		5.72	1.11				-	.67	.59	.57	.56	.55
3. Effectiveness		5.87	0.96					-	.61	.57	.50	.55
4. Efficiency		5.79	1.16						-	.64	.50	.53
5. Understanding		6.01	0.95							-	.68	.72
6. Trustworthiness		5.99	1.08								-	.86
7. Active trust		5.97	1.08									-

^a^SD: standard deviation.

Four user perceptions were positively related with e-loyalty. The effect size was highest for trustworthiness on e-loyalty directly (ß=.34), and trustworthiness was also partially mediated by active trust. Finding the website that is “easy to understand” was positively related to active trust.

Females had higher mean scores than males and other genders for e-loyalty (6.19 vs 5.80, n=133, W=2482.5, *P*=.02), trustworthiness (6.23 vs 6.02, n=133, W=2454.5, *P*=.03), and understanding (6.24 vs 5.98, n=133, W=2468, *P*=.02). However, gender identity was not found to be significantly associated with e-loyalty in the SEM and was not retained. In the SEM, increasing levels of education were associated with higher mean e-loyalty scores. Education level fully mediated the effect of visitor type (ie, client vs health care provider) on e-loyalty because health care providers generally had higher education than clients (97% [38/39] of providers had college-level education or higher vs 69.4% [75/108] of clients).

## Discussion

Overall, our study demonstrated high e-loyalty and positive user perceptions among SSR users. A new user perception, understanding, was significant in the SEM describing e-loyalty. Also, as one of the first eHealth studies to link e-loyalty with knowledge and behavioral intent outcomes, we found significant associations between e-loyalty and perceived knowledge increase for clients and providers, as well as intent to change sexual health behavior among clients.

This study contributes to the literature by presenting a parsimonious model that illustrates associations between e-loyalty to a sexual health website and user characteristics that are potentially modifiable. Understanding the nature of the inter-relationships among factors that influence e-loyalty helps to both inform the development of theory and provide insights into the processes by which we might intervene to enhance the occurrence and strength of e-loyalty. The analysis identified a set of key factors that are most amenable to intervention and ongoing monitoring. Targeting efforts toward these factors may be particularly useful considering that the way visitors currently interact with sexual health resources has limited capacity for nuance and complexity. Future research should further explore the effect of manipulating website characteristics to change such user perceptions, e-loyalty, knowledge gained, and intent to change behavior.

### E-Loyalty and Website Efficacy

The associations between e-loyalty and the survey’s other key outcomes, perceived knowledge increase, and intent to change behavior (among clients) lead us to hypothesize that e-loyalty contributes to the efficacy of this website in improving sexual health. A meta-analysis of Web-based health interventions found conflicting results regarding the relationship between intervention adherence (a reflection of e-loyalty) and behavioral outcomes but concluded they are likely to be related [19]. More longitudinal research could be done to further elucidate the relationship between e-loyalty and outcomes resulting from loyal engagement with Web-based health resources.

Our SEM provides further insights into associations between user perceptions and e-loyalty for health promotion websites, confirming previous findings and revealing new relationships.

### Trustworthiness

Trustworthiness had the strongest direct effect on e-loyalty in this model, and its effect was also partially mediated by active trust. This finding is in line with prior social marketing research that showed active trust partially mediating the effect of website credibility on behavioral intent [20]. In our study, trustworthiness and active trust were highly correlated (ρ=.86), yet both independently captured variance in e-loyalty that the other could not. This finding differs from previous health-related applications of the e-loyalty framework, in which trustworthiness was not found to be significantly related to e-loyalty for health information websites about sports injury prevention, alcohol consumption, and depression [13]. Crutzen et al hypothesized that active trust could capture all the variance in e-loyalty that could be explained by trustworthiness. We hypothesize that this differs in our study because of the inclusion of both clients and providers. On average, clients gave similar scores for trustworthiness and active trust (5.78 and 5.79, respectively), perhaps because clients’ trust in the website is directly applicable to their confidence in acting upon the information, whereas providers gave a higher score for trustworthiness than active trust (6.64 vs 5.78), which may be because their confidence in acting upon the information is not applicable as they are using the website for work. Therefore, trustworthiness may be a relevant user perception independent of active trust in the context of a website that provides information for other purposes in addition to behavior change.

There is a large body of literature recognizing the importance of trust in the use of Web-based health information [21-23]. Trust in health websites in general is known to be predicated on the reputation and respect of the organization that operates the website, the demonstration of in-depth knowledge of a variety of relevant topics, and *the delivery of clear information* [23]. For sexual health resources specifically, American studies have shown that youth are distrustful of online information about sexual health and preferred traditional forms of sexual health education, such as from parents, school, medical professionals, and friends [24,25]. However, being able to access personal expertise (ie, a traditional form of education) through a novel delivery system (an online one-on-one chat with a nurse) was considered to be a trustworthy source of information among youth in British Columbia [26]. The online presence of nurses on SSR through the “Ask a Nurse” or “Chat” functions may contribute to increased trust of both the interactive services and the static information.

SSR also has other features known to be associated with trustworthiness among specific populations. When searching for sexual health information online, youth have been shown to assess credibility largely upon a website’s domain name—dot com, dot gov, or dot org—or its association with government [24,27,28]. A study of gay and bisexual men indicated that their trust in Web-based sexual health information was based on hospital or university affiliations and also the convergence of information across multiple websites [29]. We expect that the SSR website’s clear affiliation with the BCCDC, a provincial, government-funded service provider, contributes to its trustworthiness along the path to e-loyalty.

In our model, the effect of trustworthiness on e-loyalty was also partially mediated by active trust. Active trust is characterized by a user’s confidence in acting upon information presented on a website, the only user perception in the e-loyalty model that speaks to action. As the ultimate client-oriented objective of an interactive Web-based resource such as SSR is to provide information that contributes to behavior change (eg, engaging in safer sex or getting tested for STIs), active trust is arguably the most important user perception to engender along the pathway to e-loyalty.

Increasingly, people are seeking health information on the Internet before going to health care providers, and youth are more likely than older adults both to go online first and to trust online health content [30]. As the Internet contributes to filling the sexual health information gap for youth [24] and other groups at higher risk for STIs and HIV such as MSM [31], future research could examine trust as it relates to e-loyalty toward Web-based sexual health resources across age and population subgroups.

### Understanding as an Antecedent to Active Trust

Our hypothesis that “understanding” may be related to e-loyalty was supported by the data. The SEM indicates that respondents who reported better understanding of content were more likely to have active trust; in other words, understanding was an antecedent to active trust along the pathway to e-loyalty.

These findings suggest an overlap between the theoretical frameworks of e-loyalty and eHealth literacy. As much as understanding is a component of eHealth literacy, we acknowledge that understanding content is only one small piece; eHealth literacy comprises much more than having the reading skills to understand information at an appropriate reading level [16,32]. Understanding also plays only a partial role in a new conception of “sexual health literacy,” which is envisioned as a combination of the level of sexual health knowledge one has and the capacity to employ this knowledge within sexual and social contexts [33].

Links between understanding, active trust, and e-loyalty are not yet well developed in the literature, but our findings are congruent with prior research, including a study showing strong support that understanding impacts trust beliefs and intention to use health information websites [23]. A study among adolescents suggested a connection between understanding and trust, in that a lack of capacity to analyze medical information (ie, low understanding or information-seeking skills) may hinder one’s ability to assess trustworthiness and credibility of an online source of health information [2], an essential step on the pathway to developing active trust and e-loyalty. Furthermore, a study among adults found that individuals with higher e-literacy were more likely to scrutinize and evaluate the reliability of the source and accuracy of the information to form an opinion of the site, compared with those with lower eHealth literacy [34], as measured by a validated eHealth literacy scale [35].

### Limitations

The main limitation of this study was the small sample size. Our challenges with attrition, despite offering an incentive, are reflective of the fundamental challenges of open online surveys. The use of a lottery-style incentive was selected based on cost and feasibility, informed by a literature search in 2013 [36-38], and intended to determine the most successful and economically feasible incentive style for open, Web-based surveys. The incentive was successful in oversampling Canadian respondents as intended (79% of survey respondents vs 28% of overall visitors to the website). The education level of respondents was driven up by the inclusion of health care providers and indicates that in terms of education level, the sample is neither representative of the Canadian population nor representative of youth who are a key target group for the site (70% of respondents had postsecondary education compared with 64% of the adult Canadian population) [39]. Given these limitations, further research is needed to determine how generalizable our findings are to user populations of other Web-based sexual health websites.

We also recognize the potential for bias in an open, online survey in which participants could respond more than once. In 37 (7%) instances, responses originated from the same IP address as other responses. We retained these responses in our sample, as we were unable to differentiate between duplicate responses from the same individual and different individuals accessing the survey from a shared computer or an institutional network, which may be likely among survey respondents (ie, students at a school or health care providers in a clinic).

Another limitation is evident in the selection process and the differences between respondents who completed and did not complete the e-loyalty section. As our participant recruitment method was related to e-loyalty (ie, those with e-loyalty to the site were more likely to be exposed to the banner ad or invitation email and enter the survey than the general public), and as completers of the e-loyalty section were more likely to be returning SSR users and health care providers, our findings may be biased toward visitors with higher e-loyalty. This is also evident in that returning visitors reported significantly higher e-loyalty scores, leading us to consider whether self-reported, cross-sectional surveys are an appropriate way to capture e-loyalty. Nevertheless, user perceptions from 173 unique respondents supported an SEM with adequate fit and statistically significant relationships between variables that provides useful insight into relationships between user perceptions, including the novel user perception, “understanding,” as well as knowledge and behavioral outcomes.

Finally, as a cross-sectional survey, we are not able to draw any conclusions regarding causation or determine whether the intended behavioral outcomes (seek STI and HIV testing, change sexual behavior) did, in fact, occur. Future research that includes a longitudinal component (eg, pre- and postassessment of behavior change) to validate our findings is needed.

### Conclusions

This exploratory study contributes to a growing field of literature on the applications of an e-loyalty framework to Web-based public health interventions. With the addition of a novel user perception to a theoretical framework of e-loyalty, we have shown that users’ understanding of the content affects their confidence in acting upon information presented, which in turn affects their e-loyalty to the site. Furthermore, we found positive associations between e-loyalty and measures of intervention efficacy (ie, knowledge and behavioral outcomes). We propose that e-loyalty and related user perceptions, including trustworthiness and understanding, are constructs relevant to consider in addition to efficacy measures in studies that evaluate Web-based sexual health interventions.

There is a need to further investigate how sexual health websites can deliberately engender understanding, trust, and active trust to develop e-loyalty among their users, for example, by manipulating website characteristics to change user perceptions. We echo the call to action to bring sexual health interventions to their full potential on the Internet by continuing to explore ways to increase their use and impact [40]. As the first study to adapt the e-loyalty framework specifically for a sexual health intervention, we confirm the relevance of e-loyalty as an outcome for evaluating the antecedents of the use and efficacy of Web-based public health interventions across health disciplines.

## Appendix

Multimedia Appendix 1 Structural equation modeling output (Mplus v7.2) for examining e-loyalty in an online sexual health promotion intervention.

## Abbreviations

BCCDC: BC Centre for Disease Control
CFI: comparative fit index
eHealth: electronic health
HIV: human immunodeficiency virus
IP: Internet Protocol
MSM: men who have sex with men
RMSEA: root mean squared error of approximation
SEM: structural equation modeling
SSR: SmartSexResource
STI: sexually transmitted infection
TLI: Tucker-Lewis index
