# Supplementary material for: Examining E-Loyalty in a Sexual Health Website: Cross-Sectional Study
Source: JMIR Public Health Surveill. 2017 Nov 2;3(4):e75. doi: 10.2196/publichealth.5393 (PMC5691242; doi:10.2196/publichealth.5393)
Supplement: Multimedia Appendix 1 [file publichealth_v3i4e75_app1.pdf]

```
Mplus VERSION 7.2 (Mac)
MUTHEN & MUTHEN
10/29/2014  10:47 AM

INPUT INSTRUCTIONS

  TITLE: E-loyalty data from BCCDC's SmartSexResource Visitor
Survey;

  DATA:         FILE IS ssr_loyalty.dat;
                FORMAT IS 13F11.0;


  VARIABLE: NAMES ARE type status educ gender rec
        ret overall effe effi lit tru act recp;

        USEVARIABLES ARE type educ gender rec
        ret overall effe effi lit tru act;

        MISSING ARE ALL (999);

        CATEGORICAL ARE educ gender;

  ANALYSIS:


  MODEL:    loyalty BY rec ret;
        loyalty ON type overall effe effi lit act tru;
        act ON tru lit;
        loyalty WITH educ;
        loyalty WITH gender;
        educ WITH type;

  OUTPUT:       SAMPSTAT STANDARDIZED MODINDICES;



*** WARNING
  Data set contains cases with missing on x-variables.
  These cases were not included in the analysis.
  Number of cases with missing on x-variables:  328
   1 WARNING(S) FOUND IN THE INPUT INSTRUCTIONS



E-loyalty data from BCCDC's SmartSexResource Visitor Survey;

SUMMARY OF ANALYSIS

Number of groups                                                 1
Number of observations                                         173

Number of dependent variables                                    5
Number of independent variables                                  6
```

```
Number of continuous latent variables                          1

Observed dependent variables

  Continuous
   REC         RET         ACT

  Binary and ordered categorical (ordinal)
   EDUC        GENDER

Observed independent variables
   TYPE        OVERALL     EFFE        EFFI        LIT         TRU

Continuous latent variables
   LOYALTY


Estimator                                                  WLSMV
Maximum number of iterations                                1000
Convergence criterion                                  0.500D-04
Maximum number of steepest descent iterations                 20
Maximum number of iterations for H1                         2000
Convergence criterion for H1                           0.100D-03
Parameterization                                           DELTA

Input data file(s)
  ssr_loyalty.dat

Input data format
  (13F11.0)


SUMMARY OF DATA

     Number of missing data patterns             8


COVARIANCE COVERAGE OF DATA

Minimum covariance coverage value   0.100


     PROPORTION OF DATA PRESENT


           Covariance Coverage
```

	TYPE	EDUC	GENDER	REC	RET
TYPE	1.000				
EDUC	0.850	0.850			
GENDER	0.746	0.734	0.746		
REC	0.994	0.850	0.746	0.994	

RET	0.983	0.838	0.734	0.977	0.983
ACT	0.994	0.844	0.740	0.988	0.977

Covariance Coverage

	ACT
ACT	0.994

UNIVARIATE PROPORTIONS AND COUNTS FOR CATEGORICAL VARIABLES

EDUC		
Category 1	0.034	5.000
Category 2	0.197	29.000
Category 3	0.544	80.000
Category 4	0.224	33.000
GENDER		
Category 1	0.667	86.000
Category 2	0.333	43.000

SAMPLE STATISTICS

ESTIMATED SAMPLE STATISTICS

MEANS/INTERCEPTS/THRESHOLDS

	TYPE	EDUC$1	EDUC$2	EDUC$3	GENDER$1
1	-0.639	-0.896	0.213	1.753	-1.842

MEANS/INTERCEPTS/THRESHOLDS

	REC	RET	ACT
1	-0.944	-0.496	-0.187

SLOPES

	OVERALL	EFFE	EFFI	LIT	TRU
TYPE	0.015	0.024	-0.093	0.078	0.121
EDUC	0.075	0.098	-0.197	-0.076	0.259

GENDER	-0.166	0.004	0.153	-0.286	-0.081
REC	0.217	0.109	-0.035	0.183	0.652
RET	0.306	0.129	0.060	0.142	0.459
ACT	-0.041	0.154	-0.058	0.326	0.646

CORRELATION MATRIX (WITH VARIANCES ON THE DIAGONAL)

	TYPE	EDUC	GENDER	REC	RET
TYPE	0.156				
EDUC	0.355				
GENDER	-0.268	-0.174			
REC	0.094	0.217	-0.143	0.789	
RET	0.016	0.288	0.031	0.516	0.554
ACT	-0.051	0.177	0.029	0.102	0.188

CORRELATION MATRIX (WITH VARIANCES ON THE DIAGONAL)

	ACT
ACT	0.236

THE MODEL ESTIMATION TERMINATED NORMALLY

MODEL FIT INFORMATION

Number of Free Parameters 26

Chi-Square Test of Model Fit

Value	51.183*
Degrees of Freedom	31
P-Value	0.0127

* The chi-square value for MLM, MLMV, MLR, ULSMV, WLSM and WLSMV cannot be used
for chi-square difference testing in the regular way. MLM, MLR and WLSM
chi-square difference testing is described on the Mplus website. MLMV, WLSMV,
and ULSMV difference testing is done using the DIFFTEST option.

RMSEA (Root Mean Square Error Of Approximation)

```
          Estimate                           0.061
          90 Percent C.I.                    0.029  0.090
          Probability RMSEA <= .05           0.249

CFI/TLI

          CFI                                0.945
          TLI                                0.920

Chi-Square Test of Model Fit for the Baseline Model

          Value                            411.254
          Degrees of Freedom                    45
          P-Value                           0.0000

WRMR (Weighted Root Mean Square Residual)

          Value                              0.948



MODEL RESULTS

                                                Two-Tailed
                    Estimate       S.E.  Est./S.E.    P-Value

 LOYALTY  BY
    REC                1.000      0.000    999.000    999.000
    RET                0.940      0.106      8.842      0.000

 LOYALTY  ON
    TYPE               0.107      0.112      0.961      0.336
    OVERALL            0.282      0.063      4.470      0.000
    EFFE               0.127      0.065      1.962      0.050
    EFFI               0.034      0.064      0.528      0.597
    LIT                0.080      0.095      0.834      0.404
    ACT                0.247      0.074      3.321      0.001
    TRU                0.376      0.088      4.253      0.000

 ACT      ON
    TRU                0.646      0.045     14.204      0.000
    LIT                0.326      0.055      5.963      0.000

 LOYALTY  WITH
    EDUC               0.195      0.055      3.549      0.000
    GENDER            -0.045      0.076     -0.591      0.555

 EDUC     WITH
    TYPE               0.141      0.048      2.926      0.003

 Means
    TYPE              -0.639      0.282     -2.262      0.024
```

```
 Intercepts
    REC               -0.829      0.846     -0.980      0.327
    RET               -0.388      0.488     -0.795      0.427
    ACT               -0.187      0.261     -0.717      0.474

 Thresholds
    EDUC$1            -0.896      0.982     -0.913      0.361
    EDUC$2             0.213      0.919      0.232      0.816
    EDUC$3             1.753      0.925      1.895      0.058
    GENDER$1          -1.842      0.919     -2.006      0.045

 Variances
    TYPE               0.156      0.039      3.999      0.000

 Residual Variances
    REC                0.426      0.056      7.607      0.000
    RET                0.233      0.048      4.830      0.000
    ACT                0.236      0.016     14.880      0.000
    LOYALTY            0.347      0.054      6.422      0.000


STANDARDIZED MODEL RESULTS


STDYX Standardization

                                                    Two-Tailed
                    Estimate       S.E.  Est./S.E.    P-Value

 LOYALTY  BY
    REC                0.880      0.028     31.540      0.000
    RET                0.920      0.022     41.209      0.000

 LOYALTY  ON
    TYPE               0.035      0.036      0.963      0.336
    OVERALL            0.260      0.057      4.523      0.000
    EFFE               0.101      0.051      1.988      0.047
    EFFI               0.033      0.062      0.528      0.598
    LIT                0.063      0.073      0.856      0.392
    ACT                0.219      0.064      3.421      0.001
    TRU                0.337      0.072      4.666      0.000

 ACT      ON
    TRU                0.653      0.038     16.992      0.000
    LIT                0.290      0.044      6.534      0.000

 LOYALTY  WITH
    EDUC               0.331      0.089      3.722      0.000
    GENDER            -0.076      0.129     -0.593      0.553

 EDUC     WITH
    TYPE               0.355      0.094      3.783      0.000

 Means
```

TYPE	-1.616	0.669	-2.413	0.016
**Intercepts**				
REC	-0.604	0.587	-1.029	0.304
RET	-0.314	0.383	-0.822	0.411
ACT	-0.174	0.240	-0.728	0.467
**Thresholds**				
EDUC$1	-0.896	0.982	-0.913	0.361
EDUC$2	0.213	0.919	0.232	0.816
EDUC$3	1.753	0.925	1.895	0.058
GENDER$1	-1.842	0.919	-2.006	0.045
**Variances**				
TYPE	1.000	0.000	999.000	999.000
**Residual Variances**				
REC	0.226	0.049	4.599	0.000
RET	0.153	0.041	3.730	0.000
ACT	0.206	0.028	7.480	0.000
LOYALTY	0.238	0.038	6.260	0.000

STDY Standardization

	Estimate	S.E.	Est./S.E.	Two-Tailed P-Value
**LOYALTY BY**				
REC	0.880	0.028	31.540	0.000
RET	0.920	0.022	41.209	0.000
**LOYALTY ON**				
TYPE	0.035	0.036	0.963	0.336
OVERALL	0.234	0.052	4.513	0.000
EFFE	0.105	0.053	1.991	0.047
EFFI	0.028	0.053	0.528	0.598
LIT	0.066	0.077	0.856	0.392
ACT	0.219	0.064	3.421	0.001
TRU	0.311	0.067	4.621	0.000
**ACT ON**				
TRU	0.603	0.042	14.211	0.000
LIT	0.304	0.047	6.449	0.000
**LOYALTY WITH**				
EDUC	0.331	0.089	3.722	0.000
GENDER	-0.076	0.129	-0.593	0.553
**EDUC WITH**				
TYPE	0.355	0.094	3.783	0.000
**Means**				
TYPE	-1.616	0.669	-2.413	0.016

```
 Intercepts
    REC               -0.604      0.587     -1.029      0.304
    RET               -0.314      0.383     -0.822      0.411
    ACT               -0.174      0.240     -0.728      0.467

 Thresholds
    EDUC$1            -0.896      0.982     -0.913      0.361
    EDUC$2             0.213      0.919      0.232      0.816
    EDUC$3             1.753      0.925      1.895      0.058
    GENDER$1          -1.842      0.919     -2.006      0.045

 Variances
    TYPE               1.000      0.000    999.000    999.000

 Residual Variances
    REC                0.226      0.049      4.599      0.000
    RET                0.153      0.041      3.730      0.000
    ACT                0.206      0.028      7.480      0.000
    LOYALTY            0.238      0.038      6.260      0.000


STD Standardization

                                                    Two-Tailed
                    Estimate       S.E.  Est./S.E.    P-Value

 LOYALTY  BY
    REC                1.208      0.124      9.733      0.000
    RET                1.135      0.093     12.263      0.000

 LOYALTY  ON
    TYPE               0.089      0.092      0.963      0.336
    OVERALL            0.234      0.052      4.513      0.000
    EFFE               0.105      0.053      1.991      0.047
    EFFI               0.028      0.053      0.528      0.598
    LIT                0.066      0.077      0.856      0.392
    ACT                0.204      0.059      3.441      0.001
    TRU                0.311      0.067      4.621      0.000

 ACT      ON
    TRU                0.646      0.045     14.204      0.000
    LIT                0.326      0.055      5.963      0.000

 LOYALTY  WITH
    EDUC               0.331      0.089      3.722      0.000
    GENDER            -0.076      0.129     -0.593      0.553

 EDUC     WITH
    TYPE               0.141      0.048      2.926      0.003

 Means
    TYPE              -0.639      0.282     -2.262      0.024
```

```
 Intercepts
    REC               -0.829      0.846     -0.980      0.327
    RET               -0.388      0.488     -0.795      0.427
    ACT               -0.187      0.261     -0.717      0.474

 Thresholds
    EDUC$1            -0.896      0.982     -0.913      0.361
    EDUC$2             0.213      0.919      0.232      0.816
    EDUC$3             1.753      0.925      1.895      0.058
    GENDER$1          -1.842      0.919     -2.006      0.045

 Variances
    TYPE               0.156      0.039      3.999      0.000

 Residual Variances
    REC                0.426      0.056      7.607      0.000
    RET                0.233      0.048      4.830      0.000
    ACT                0.236      0.016     14.880      0.000
    LOYALTY            0.238      0.038      6.260      0.000


R-SQUARE

    Observed                                        Two-Tailed
Residual
    Variable        Estimate       S.E.  Est./S.E.    P-Value
Variance

    REC                0.774      0.049     15.770      0.000
    RET                0.847      0.041     20.605      0.000
    ACT                0.794      0.028     28.876      0.000

     Latent                                         Two-Tailed
    Variable        Estimate       S.E.  Est./S.E.    P-Value

    LOYALTY            0.762      0.038     20.062      0.000


QUALITY OF NUMERICAL RESULTS

     Condition Number for the Information Matrix
0.241E-03
       (ratio of smallest to largest eigenvalue)


MODEL MODIFICATION INDICES

NOTE:  Modification indices for direct effects of observed dependent
variables
regressed on covariates and residual covariances among observed
dependent
variables may not be included.  To include these, request MODINDICES
(ALL).
```

```
Minimum M.I. value for printing the modification index    10.000

                                   M.I.     E.P.C.  Std E.P.C.
StdYX E.P.C.

No modification indices above the minimum value.



     Beginning Time:  10:47:13
        Ending Time:  10:47:13
       Elapsed Time:  00:00:00
```

MUTHEN & MUTHEN
3463 Stoner Ave.
Los Angeles, CA 90066

Tel: (310) 391-9971
Fax: (310) 391-8971
Web: www.StatModel.com
Support: Support@StatModel.com

Copyright (c) 1998-2014 Muthen & Muthen
